# Case report: Successful pregnancy complicated with non-cirrhotic portal hypertension in a lady who suffered from postpartum hemorrhage previously

**DOI:** 10.1097/MD.0000000000034659

**Published:** 2023-09-29

**Authors:** Xiaoxi Niu, Yanmin Gong, Xia Luo

**Affiliations:** a Department of Obstetrics and Gynecology, Shandong University Qilu Hospital, Jinan, Shandong, China.

**Keywords:** case report, cesarean section, hypersplenism, non-cirrhotic portal hypertension, postpartum hemorrhage

## Abstract

**Rationale:**

Non-cirrhotic portal hypertension (NCPH) is characterized by the absence of cirrhotic modification of the liver and the patency of the portal and hepatic veins. When compared to the general population, NCPH is associated with an increased risk of maternal and perinatal morbidity and mortality during pregnancy. NCPH was present in the majority (74.1%) of pregnant women with portal hypertension. One (25%) out of every 4 pregnancies was complicated by variceal hemorrhage while pregnant. So far, there is still no consensus in the world about the treatment of this rare condition.

**Patient concerns:**

We have specifically illustrated a rare instance where the patient was diagnosed with NCPH and hypersplenism at the age of 8 and experienced a 3 L massive hemorrhage during labor induction as a result of her first pregnancy loss due to hypertension.

**Diagnoses and Interventions:**

The diagnosis of threatened preterm labor with cervical dilatation, gestational diabetes mellitus, massive splenomegaly with hypersplenism, portal vein hypertension, and parenchymal damage of kidney with impaired renal function led to the cesarean delivery of the second pregnancy at 29^+3^ weeks gestation without splenectomy after been evaluated by multispecialty team.

**Outcomes:**

She and her child were both in generally good condition 3 months after the operation.

**Lessons:**

Preconception counseling, ongoing follow-up, and monitoring are crucial in pregnant women with NCPH. A multidisciplinary team approach, with timely intervention and intensive monitoring, can help achieve optimal maternal–perinatal outcomes in pregnancies complicated with portal hypertension. Our case provided a successful treatment, but more guidelines for the management of NCPH are needed.

## 1. Introduction

The absence of cirrhotic alteration of the liver and the patency of the portal and hepatic veins are the characteristics of non-cirrhotic portal hypertension (NCPH).^[1]^ Women with non-cirrhotic portal hypertension frequently become pregnant. NCPH affects 3% to 5% of the population worldwide.^[2]^ Women of childbearing age make up 15% of NCPH patients.^[1]^ When compared to the general population, portal hypertension (PH) is linked to a higher risk of maternal and perinatal morbidity and mortality during pregnancy, including variceal bleeding, abortion, fetal growth restriction, preterm birth, stillbirth, postpartum hemorrhage, and the prevalence of cesarean delivery.^[3]^

It is unidentified how and when to terminate the pregnancy because there are scant and disparate reports on pregnancies in women with NCPH. There have been reports of combined caesareans and splenectomy during pregnancies with PH.^[4]^ However, not all individuals who are pregnant must follow this surgery. Specifically, we have shown a rare case who received an uncommon diagnosis of NCPH and hypersplenism at the age of 8 and suffered a 3 L major bleed during labor induction as a result of the stillbirth caused by hypertension in her first pregnancy. The second pregnancy was delivered via cesarean section at 29^+3^ weeks gestation without splenectomy with the diagnosis of threatened preterm labor with cervical dilatation, gestational diabetes mellitus, massive splenomegaly with hypersplenism, portal vein hypertension, and parenchymal damage of kidney with impaired renal function.

## 2. Case presentation

A 32-year-old woman was admitted to the medical unit of Shandong University Qilu Hospital in Jinan, Shandong, China in April 2022 for the diagnostic workup of threatened preterm labor with cervical dilatation, gestational diabetes mellitus, massive splenomegaly with hypersplenism, portal vein hypertension, and parenchymal damage of kidney with impaired renal function. Despite experiencing nausea and dyspnea, she did not throw up. An electronic cardiotocography instrument recorded at least 2 regular, painful uterine contractions in 20 minutes, each of which lasted for at least 30 seconds. She denied having any chest or heart problems, fetal membranes rupturing, or vaginal bleeding.

She came to our hospital for the first time after being referred by a nearby hospital. She encountered an incident of hematemesis when she was 8 years old, according to her retrospective history, for which she underwent extensive examinations, including a blood test, abdominal ultrasound, and an upper gastrointestinal (GI) endoscopy. She was later identified as having significant splenomegaly, hypersplenism, and idiopathic portal hypertension with no recognized explanation. Nearly every year after that, she underwent blood transfusions to treat her thrombocytopenia brought on by hypersplenism. Prior to becoming pregnant, she had previously suffered from acute esophageal varices bleeding on multiple occasions; nevertheless, the local hospital’s attempt at endoscopic variceal ligation failed. At her first pregnancy, intraamniotic injection of ethacridine lactate for second trimester (28 weeks gestation) termination was conducted because of stillbirth for preeclampsia. She had a massive bleed of 3 L during induction of labor with no episode of GI bleeding.

At this visit, her full blood count showed: hemoglobin 77 g/L, white blood cell 3310/mm^3^, platelets 45,000/mm^3^, reticulocyte count 3.88% and no evidence of coagulopathy. Other investigations measured: blood urea nitrogen 8.2 mmol/L, creatinine 102 umol/L, uric acid 502 umol/L, lactate dehydrogenase 235 U/L with normal biochemistry. Urine analysis was normal. Another testing such as antinuclear antibodies, bone marrow aspiration, Coombs test, blood sugar, and lipid profile were normal.

**Figure 1.**
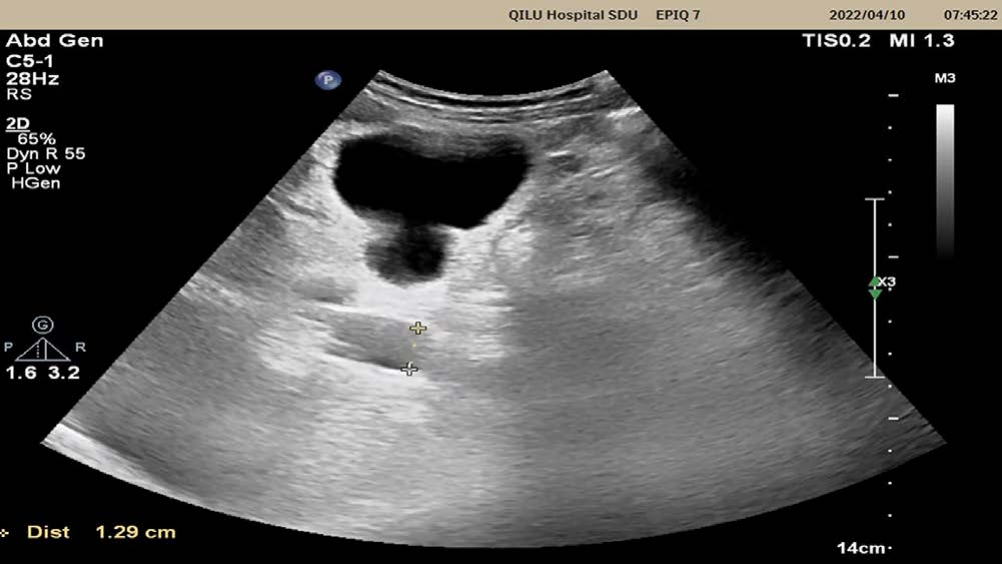
Ultrasound depicted a 13 mm dilated portal vein.

An abdominal ultrasound revealed extensive parenchymal damage to both kidneys, several kidney cysts, a 23.6 cm enlarged spleen with a dilated splenic vein and a 13 mm dilated portal vein (Fig. 1), but no ascites. The ultrasonography excluded cirrhosis and clearly demonstrated PH. Upon abdominal examination, a normal-sized liver and palpable spleen were found 10 cm below the left costal cartilage. The uterus matched the 28-week gestational period. By using a speculum to examine the membrane, 1 cm of it could be seen. The area around the uterine fundus was sensitive. The uterus was mushy, and no discernible contractions could be felt. According to obstetric ultrasound measurements, the internal os of the cervical cavity dilated by 3.1 cm, and the prolapsed membranes reached the external os. An ultrasound showed that the fetus was normal and concordant with gestational age. In addition, cardiac ultrasound detected dilated cardiomyopathy and mild pulmonary hypertension.

Patients were made aware of the dangers related to variceal bleeding, spleen rupture, and postpartum hemorrhage. When combined with dexamethasone, tocolytic drugs such intravenous magnesium sulfate for 48 hours demonstrated a fetal neuroprotective effect in reducing cerebral palsy in premature newborns. Her blood parameters were checked often. During hospitalization, a gradual decline in fibrinogen levels was observed.

The patient was taken to the operating room with a presumed diagnosis of splenomegaly, thrombocytopenia, and coagulopathy 6 days after check-in. Obstetric and general surgeons were present when a midline incision C-section under general anesthetic was performed. A healthy single baby girl weighing 1100 g was delivered via cesarean section; the infant had an Apgar score of 6 (heart rate 2, respiratory effort 0, muscular tone 1, reflex irritability 1, and color 2) at birth. In response to resuscitation, the baby’s 5-minutes Apgar score was 7 (heart rate 2, respiratory effort 1, muscular tone 1, reflex irritability 1, and color 2). 46 days after birth, the infant was taken to the NICU and then released. The entire procedure took 2 hours, and there was a 400 mL intraoperative blood loss. 360 mL of plasma, 2 units of apheresis platelets, and 4 units of packed red blood cells were used to replace the lost blood. No signs of placental abruption or severe upper abdominal hemorrhage were present. After weighing the risks and advantages of surgery, a splenectomy was avoided because there was no evidence of intraperitoneal bleeding. Throughout the surgery, the mother’s blood pressure and heart rate stayed within normal limits.

**Figure 2.**
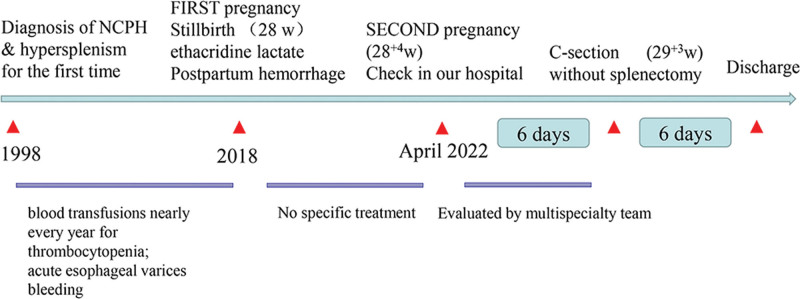
The timeline of patient’s history.

The patient had a successful recovery following surgery and was released on the sixth postoperative day (Fig. 2). Recovery from surgery was uneventful for both mother and child. There were no inherited defects in the infant.

Three months following the operation, we conducted telephone follow-up because the patient refused to come back to the hospital. Her platelets were 75,000/mm^3^ at the time of her initial evaluation, and she and her child were both in generally good condition. She intended to undergo a splenectomy because the spleen had reduced in size.

## 3. Discussion

Non-cirrhotic PH is not common in women of reproductive age. Andrade F et al published thorough data demonstrating that pregnancy in women with NCPH typically has a satisfactory maternal and fetal outcome despite a substantial incidence of PH related complications.^[1]^ This is because reports on pregnancy with NCPH are sparse and heterogeneous.^[4–7]^ Pregnancy complications are more frequent and severe in cirrhotic PH than in non-cirrhotic PH because the severity of the liver disease is closely correlated with poor maternal and neonatal outcomes in PH.^[7]^

Unfavorable pregnancy outcomes are linked to the catastrophic PH complication of GI hemorrhage. According to reports, 78% of pregnant women who had esophageal varices diagnosed with PH before becoming pregnant will experience bleeding.^[8]^ The second, third trimester, and second stage of labor may provide the highest risks for active bleeding. It was advised that all expectant women with esophageal varices undergo endoscopic sclerotherapy or a decompression procedure in the early second trimester or before becoming pregnant.^[9]^ Beta blockers are linked to fetal growth restriction and bradycardia, despite the fact that they lessen portal pressure and variceal bleeding.^[10]^ Although our patient had less severe acute bleeding before becoming pregnant, the endoscopic variceal ligation procedure was not being performed successfully in the nearby hospital. Therefore, bleeding during labor is highly likely.

Manjunath Maruti Pol reported a patient that underwent cesarean section owing to failed induction of labor combined with splenectomy in pregnancy with PH at 38 weeks gestation.^[4]^ She has undergone endoscopic sclerotherapy for esophageal varices in the past. The patient and her child had an uneventful postoperative recovery and were discharged on the ninth postoperative day. The author also suggested management for treatment in pregnancy with PH in varied situation.

Keepanasseril A^[11]^ reported an observational study which examined the maternal and perinatal outcomes of pregnant women with NCPH over a 9-year period at a South Indian tertiary hospital. Of 108 pregnancies with PH, 74.1% had NCPH, with pancytopenia or splenomegaly as the presenting symptoms in over half of the cases. Variceal bleeding was the most common complication in women with NCPH, with 25% of pregnancies affected and 3 cases experiencing massive bleeding. Preterm birth was the most common obstetric complication, and there was 1 maternal death due to severe sepsis, acute kidney injury, and disseminated intravascular coagulation following massive variceal bleeding. The study suggests that a multidisciplinary team approach, with timely intervention and intensive monitoring, can help achieve optimal maternal-perinatal outcomes in pregnancies complicated with PH. Similarly, a multispecialty team of physicians, including an obstetrician, general surgeons, gastroenterologists, nephrologists, hematologists, and cardiologists, assessed our case. Due to pulmonary hypertension, poor blood coagulation, postpartum hemorrhage from a prior pregnancy, and a medical history of variceal bleeding without endoscopic therapy, vaginal birth was not advised. Although upper GI endoscopy during the second trimester has been suggested in the literature, the usage is limited to fetal hypoxia brought on by sedation or placement.^[11]^ Furthermore, this patient experienced severe uterine contractions, making UGIE inappropriate. Overall, the maternal and perinatal complications were comparable to the previous studies.

The safety and viability of postpartum splenectomy or combination cesarean with splenectomy were also discussed with our patient. Because postsplenectomy patients may be at risk for problems including infection and postpartum hemorrhage that may be brought on by a lengthy procedure or extensive excision, it has been suggested that splenectomy is not necessary in this circumstance.^[4]^ Additionally, the patient had no desire for a single procedure.

At 29^+3^ weeks gestation, a cesarean delivery was performed, and a splenectomy was advised to be done 3 to 6 months following delivery. During and following surgery, something should be kept in mind. Loss of operating space in the abdomen is brought on by the uterus during pregnancy and the enlarged spleen. Megaspleen huge size and friability make it easily rupture with vigorous manipulation. Therefore, delicate tissue handling is a crucial and significant component of surgical performance. Between 7% and 26% of pregnancies with PH experience postpartum bleeding, which is typically brought on by hypersplenism-related coagulopathy and thrombocytopenia. In our example, a postpartum hemorrhage was prevented by improving the blood poor coagulation during surgery.

## 4. Conclusion

Counseling prior to conception, regular follow-up, and monitoring are essential in these circumstances. Pregnant women with NCPH should be moved as soon as possible to a hospital with a history of providing acute and critical care. There are currently no clear protocols for treating NCPH during pregnancy, which raises the risk of negative outcomes for both the mother and the fetus. Further studies are also required to analyze more cases to evaluate the feasibility of different methods of pregnancy termination in patients with NCPH and how to deal with the maternal and perinatal outcomes.

## Acknowledgments

The authors are thankful to our patient for her informed consent, the Ethics Review Committee of our hospital.

## Author contributions

**Conceptualization:** Yanmin Gong, Xia Luo.

**Formal analysis:** Xiaoxi Niu.

**Funding acquisition:** Xia Luo.

**Project administration:** Yanmin Gong.

**Supervision:** Xia Luo.

**Writing – original draft:** Xiaoxi Niu.

**Writing – review & editing:** Yanmin Gong, Xia Luo.
